# Generating actionable insights from free-text care experience survey data using qualitative and computational text analysis: A study protocol

**DOI:** 10.12688/hrbopenres.13606.1

**Published:** 2022-09-12

**Authors:** Daniela Rohde, Mona Isazad Mashinchi, Nina Rizun, Dritjon Gruda, Conor Foley, Rachel Flynn, Adegboyega Ojo

**Affiliations:** 1 National Care Experience Programme, Health Information and Quality Authority, Dublin, Ireland; 2 Maynooth University School of Business, Maynooth University, Maynooth, Co Kildare, Ireland; 3 Department of Informatics in Management, Gdansk University of Technology, Gdansk, Poland; 4 School of Public Policy and Administration (SPPA), Carleton University, Ottawa, K1S 5B6, Canada

**Keywords:** quality of health care, patient satisfaction, qualitative research, text analysis, information science

## Abstract

**Introduction:**The National Care Experience Programme (NCEP) conducts national surveys that ask people about their experiences of care in order to improve the quality of health and social care services in Ireland. Each survey contains open-ended questions, which allow respondents to comment on their experiences. While these comments provide important and valuable information about what matters most to service users, there is to date no unified approach to the analysis and integration of this detailed feedback. The objectives of this study are to analyse qualitative responses to NCEP surveys to determine the key care activities, resources and contextual factors related to positive and negative experiences; to identify key areas for improvement, policy development, healthcare regulation and monitoring; and to provide a tool to access the results of qualitative analyses on an ongoing basis to provide actionable insights and drive targeted improvements.

**Methods:**Computational text analytics methods will be used to analyse 93,135 comments received in response to the National Inpatient Experience Survey and National Maternity Experience Survey. A comprehensive analytical framework grounded in both service management literature and the NCEP data will be employed as a coding framework to underpin automated analyses of the data using text analytics and deep learning techniques. Scenario-based designs will be adopted to determine effective ways of presenting insights to knowledge users to address their key information and decision-making needs.

**Conclusion:**This study aims to use the qualitative data collected as part of routine care experience surveys to their full potential, making this information easier to access and use by those involved in developing quality improvement initiatives. The study will include the development of a tool to facilitate more efficient and standardised analysis of care experience data on an ongoing basis, enhancing and accelerating the translation of patient experience data into quality improvement initiatives.

## Introduction

Patient experiences are central to the delivery of quality, person-centred healthcare and an important predictor of outcomes ^1– 3^. Patient experiences have been linked with clinical effectiveness and patient safety, including medication and treatment adherence, health-promoting behaviour, healthcare use, adverse events and mortality ^2, 4^. Collecting patient experience data, including through surveys, feedback and complaints, provides unique insights into the quality of healthcare, and can inform quality improvement efforts ^5^. Quality improvement interventions, in turn, have successfully improved patient experiences. A recent systematic review of hospital quality improvement interventions found that of 21 included studies, 20 reported improvements in patient experiences resulting from quality improvement strategies ^6^. Improvement efforts, however, are not universally effective ^7, 8^, with some studies noting barriers in terms of the extent to which patient experiences are used to inform quality improvement interventions ^6, 9^, While there has been growing interest in the analysis of patient comments and complaints to inform the design of safer and more patient-centred healthcare, a limitation of current comment and complaint handling systems is that they are mainly designed to deal with complaints on a case-by-case basis ^10^, limiting the extent to which they can be used to inform service-level improvements.

While quantitative care experience surveys are useful in facilitating the collection of large samples and standardised data, they may lack sufficient detail on the precise aspects of experiences that are important to patients and may not be ideally suited to identifying specific areas where quality improvement efforts could have the greatest impact ^5, 9, 11^. Open formats, which allow patients to explain their experiences and provide contextualised feedback and suggestions for change and improvement, are essential for the collection of detailed, patient-centred data ^12^. Qualitative data may be more interesting, meaningful and relevant to clinicians and ward staff, providing more comprehensive and in-depth understanding of care experiences and identifying new insights and areas for action ^8, 9, 11^. Negative comments in particular provide specific details and information that can be used to inform plans to improve patient experiences ^11^. Qualitative data, however, are more time-consuming and costly to collect and analyse ^13^. There is to date no unified approach to the analysis and integration of the detailed feedback received in response to open-ended questions included on routinely conducted care experience surveys. Supporting healthcare providers to analyse and use comments and complaints in a more efficient and standardised manner could have important impacts on the quality of healthcare ^10^.

### The National Care Experience Programme

The Committee on the Future of Healthcare’s Sláintecare report, which outlines a cross-party vision for Ireland’s health and social care services and policy, highlights that patients’ needs must ‘come first in driving safety, quality and the coordination of care’ ^14^. The Irish National Standards for Safer Better Healthcare aim to ‘create a basis for improving the quality and safety of healthcare services by identifying strengths and highlighting areas for improvement’ ^15^. The National Care Experience Programme (NCEP) seeks to improve the quality of health and social care services in Ireland by asking people about their experiences of care and acting on their feedback. The NCEP collects a large volume of qualitative data, received in response to open-ended care experience survey questions. These questions allow service-users to describe the care they received in their own words, including aspects that are not captured by other survey questions. The comments provide a rich source of information on patients’ experiences with healthcare. Previous analyses of these data, however, have tended to focus on specific, limited areas or topics, such as women’s experiences of initiating infant feeding or older people’s experiences in the emergency department ^16, 17^. The volume of comments makes it difficult to extract all key themes and areas of experience that could be used to inform quality improvement plans and changes in practice and policy on a larger scale.

To date, 93,135 comments have been received from participants in the National Inpatient Experience Survey and National Maternity Experience Survey. As the NCEP expands to include additional surveys, the amount of qualitative data collected will increase substantially. This study will provide important, in-depth insights into areas of good experience and areas requiring improvement across acute hospital and maternity services, based on secondary analysis of detailed first-hand accounts from patients and women using these services.

### Aims and objectives

This study aims to employ a systematic, computational approach to the categorisation and analysis of large volumes of qualitative data from Irish national care experience surveys in order to extract key themes and areas of experience, which will in turn inform national efforts to improve health and social care delivery, policy and practice. A dashboard will be developed to make the findings available and useable to those with the power and responsibility to act on them. A second aim of this study is to inform the development of a tool that will facilitate more efficient and standardised processing and analysis of care experience data received by healthcare services and other organisations.

The objectives of the study are to:

- Analyse qualitative responses to the National Care Experience Programme surveys to determine the key care activities, resources and contextual (ARC) factors related to positive and negative care experiences across different patient and demographic groups
- Identify key areas for improvement, policy development, healthcare regulation and monitoring, as well as possible quality improvement initiatives
- Provide quality managers, practitioners, and other stakeholders with a tool to access the results of qualitative analyses on an ongoing basis to provide actionable insights and drive targeted improvements.

## Protocol

### Study design and data sources

This study will involve secondary analysis of data from the National Inpatient Experience Survey and National Maternity Experience Survey. The study commenced on 1 April 2022, and will run for 24 months. National Inpatient Experience Survey and National Maternity Experience Survey data were requested for this research purpose from the National Care Experience Programme by completing a data access request form. 

***National Inpatient Experience Survey.*** The National Inpatient Experience Survey (NIES) is a national, repeat cross-sectional survey of inpatient experiences in all 40 public acute hospitals in Ireland ( Table 1). The NIES was conducted annually from 2017 to 2019, cancelled in 2020 as a result of the COVID-19 pandemic, and resumed in 2021. All patients aged 16 years and above, who spend at least 24 hours in one of the participating hospitals and who are discharged during the survey month are eligible to participate, with response rates ranging from 42–51%. The survey asks patients about their experiences in hospital from admission to discharge. Overall experience of care is measured on a scale from 0 (very poor) to 10 (very good). Three open-ended questions are included, which ask patients if there was anything particularly good about their hospital care, if there was anything that could be improved, and if they have any other comments or suggestions. In 2021, the last question was amended to ask if patients had any other comments about how the COVID-19 pandemic affected their care. Since 2017, 50,196 patients have participated in the survey, with a total of 86,239 comments received in response to the open-ended questions.

**Table 1.** Overview of the National Inpatient Experience Survey and National Maternity Experience Survey.

	National Inpatient Experience Survey	National Maternity Experience Survey
**Survey years**	2017–2019, 2021	2020
**Number of hospitals/services**	All 40 public acute hospitals in Ireland	All 19 maternity hospitals/units in Ireland; National Home Birth Service
**Area of care**	Acute hospital inpatient care	Maternity care
**Stages of care**	Admission to hospital; care on the ward; examinations, diagnosis and treatment; discharge or transfer; other aspects of care; care during the pandemic (2021 survey)	Care while pregnant; care during labour and birth; care in hospital after the birth; specialised care; feeding; care at home after the birth
**Overall care**	Rated on a scale from 0 (very poor) to 10 (very good)	Rated on a scale from 0 (very poor) to 10 (very good)
**Eligibility criteria**	All patients aged 16+ years, with a postal address in Ireland, who spent 24+ hours in a participating hospital and were discharged during the survey month (May or September). Excluding day care, maternity, psychiatric, paediatric and other specialist services.	All women aged 16+, with a postal address in Ireland, who gave birth in one of Ireland’s maternity hospitals/units or had a home birth during the survey months (October or November). Excluding women who experienced a pregnancy loss, concealed pregnancy, or whose baby was taken into care.
**Total participants**	50,196	3,205
**Response rates**	42–51%	50%
**Number of survey questions**	61–67	68
**Number of free-text questions**	3	3
**Total number of comments received**	86,239	6,896

***National Maternity Experience Survey.*** The National Maternity Experience Survey is a national survey of maternity care experiences, which was conducted for the first time in 2020 ( Table 1). The survey asks women about their maternity care experience from antenatal care to care during labour and birth and care at home after the birth. Overall experience of care is measured on a scale from 0 (very poor) to 10 (very good). Three open-ended questions ask women if there was anything particularly good about their maternity care, if there was anything that could be improved, and if there were any other important parts of their maternity care experience that were not covered by the questions in the survey. Women aged 16 years and above, who gave birth in one of Ireland’s 19 maternity hospitals or units, or who had a home birth in October or November 2019, were eligible to participate. In 2020, 3,205 women participated, representing a response rate of 50%. A total of 6,896 comments were received in response to the three open-ended questions.

### Data analysis

***Development of an analytical framework.*** In-depth analysis of qualitative data requires an overarching theoretical or conceptual foundation. This is often operationalised as an analytical framework, comprised of a set of codes or categories that are used to organise and manage unstructured, free-text data ^18^. To explore issues related to both positive and negative experiences of patients in acute hospital and maternity care contexts, the analysis will be grounded in the Service (Dominant) Logic theoretical framework ^19^. In this framework, the notion of patient or maternity care as a service involves a process that facilitates the realisation of value (e.g. safe delivery) from the patient’s or mother’s perspective ^20^. Patient experiences are conceptualised as the outcome of the interactions of the patient with systems, administrative and clinical processes, and with the different types of staff providing care ^20^. The use of Ordenes *et al.*’s Activities, Resources, and Context (ARC) framework enables a focus on information about the different processes or activities and resources (human, material and non-material) characterising the patient interaction, as well as the context of that interaction (i.e. the personal situation of the patient or woman and the circumstances of their care) ^20^.

**Figure 1.**
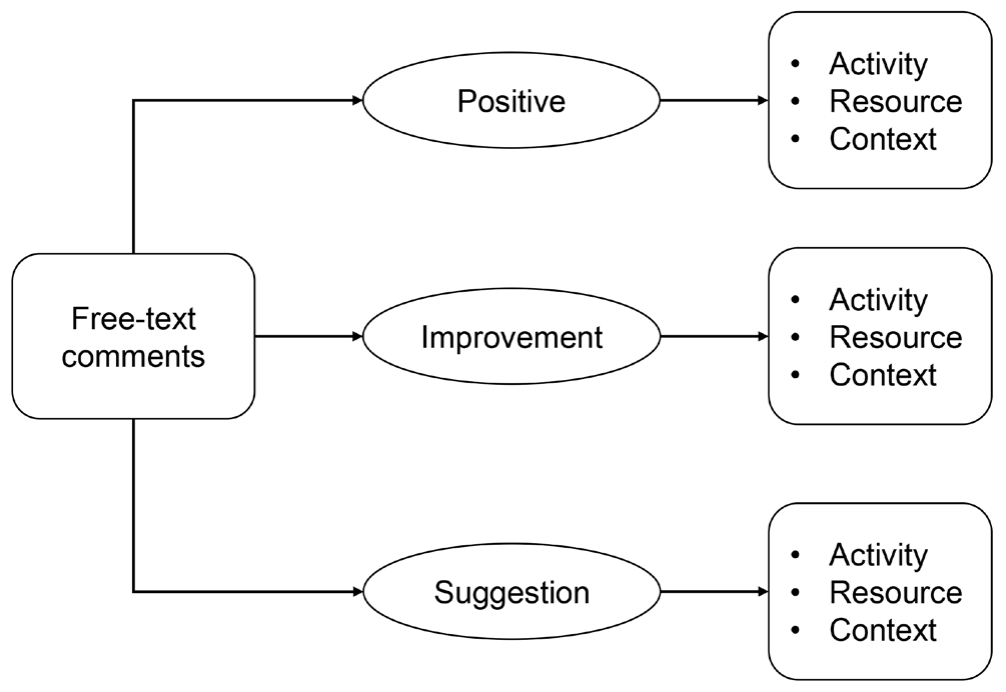
The Activity-Resource-Context Framework.

For this study, activities refer to specific patient touchpoints; resources comprise the care staff, administrative and medical processes, equipment and facilities utilised when providing care to patients and women; while contexts cover the different types of situational and personal circumstances of patients and women when receiving care. The NCEP stages of care ( Table 1) will be used as top-level categories for activities, while the Systematized Nomenclature of Medicine (SNOMED) healthcare terminology will be adopted in defining the taxonomy for resources. The taxonomy for contexts will be developed inductively from the qualitative data. The resulting Activity-Resource-Context Framework is shown in Figure 1.

***Exploratory data analysis.*** The initial exploratory phase of this study will entail automated analysis of the qualitative data using computational text analytics and predictive machine learning techniques, including structural topic models and predictive modelling. The exploratory analyses will help to refine the analytical framework by uncovering additional important concepts related to activities, resources and contexts contained within the comments but not captured in the analytical framework, and will inform the more detailed subsequent analysis of annotated data.

**Structural topic models**

Structural topic models of the qualitative data will be developed to uncover important themes and relationships between these themes and demographic (age, sex, ethnicity) and organisational (e.g. hospital size) factors. Topic modelling is a statistical approach used to extract the hidden semantics that occur in a collection of documents or other textual data. The Latent Dirichlet Allocation (LDA) technique is one of the most notable approaches for unsupervised topic modelling. LDA defines themes in the textual data as distributions over a vocabulary of words that represent semantically interpretable topics. The meaning of those topics is based on the emergent quality of the relationship between words ^21^. Structural topic modelling (STM) is an extension of the LDA framework that has previously been adopted in qualitative patient experience studies ^22– 24^. STM extends LDA, which only analyses information “about the content of the text”, by also analysing information about the unstructured data itself ^25^. Consequently, STM facilitates the exploration of relationships between themes in the qualitative data and demographic and organisational factors in a systematic manner and on a larger scale than would be possible using traditional qualitative analysis techniques.

**Predictive modelling with Random Forest**

Predictive modelling, using an ensemble of machine learning algorithms such as Random Forest and Support Vector Machines, will then be used to determine which of the extracted themes or topics most strongly influence overall care experience ratings. The Random Forest algorithm combines multiple decision trees and relies on each random sample to build each decision tree, reducing the risk of overfitting. As a classifier, Random Forest performs an implicit feature selection, using a subset of "strongest variables" for the classification only to reach the best performance on high dimensional data ^26^. The outcome of this implicit feature selection of the Random Forest algorithm can be determined and visualised as a concept of variable importance (Gini Importance) ^27^. The concept of variable importance here is synonymous with topic importance regarding the overall patient experience rating. The predictive model will determine the overall care experience rating (ranging from poor to very good) that is most probable for a specific distribution of topics across a comment. The model will also identify the relative importance of the latent topics or issues characterising the data.

### Pilot and full dataset annotation

Annotation of the datasets using the analytical framework will be completed in two stages. The pilot phase will test and refine the analytical framework to reduce ambiguity or inconsistency in the developed ARC taxonomies, using a random sample of comments (~10%) from the two datasets. Two experienced qualitative researchers will create high-quality annotations, which will serve as training data for automatic annotation of the full datasets by a machine or deep learning model in the next stage. Given the anticipated use of the annotated dataset, we will aim for strong interrater reliability (>0.8). Computer-based qualitative research software (MaxQda, as well as an in-house developed application) will be used for the pilot annotation task.

The full annotation of the dataset will involve the development of a machine or deep learning model to automatically identify and label ARC entities in the comments, due to the significant amount of time and resources required to reliably manually annotate over 90,000 free-text feedback comments. A variety of features from the manually coded datasets from the pilot phase will be extracted, including Part-of-Speech (PoS) tags, trigger terms identified by annotators for ARC terms in the text, and multidimensional vector representations of the ARC terms using combinations of embeddings. We frame the process of automatic ARC annotation of comments as Named Entity Recognition (NER) and (ARC) Relation Extraction problems ^28, 29^. A Long Short Term Memory (LSTM) based deep-learning model will be adopted for ARC NER and relation extraction. Specifically, we will explore the use of bidirectional LSTM, LSTM-Conditional Random Field and the n-Gram Convolutional Neural Nets (GRAM-CNN) models ^30, 31^.

***ARC pattern mining.*** Following the complete annotation of qualitative data, important or frequently occurring ARC patterns or events associated with both positive and negative experiences will be identified. An Association Rule and Sequence Mining, such as the Apriori algorithm, will be used to extract ARC patterns in the annotated datasets. The Apriori algorithm is a popular data mining algorithm for mining frequent item sets and associated association rules from a dataset. The extracted patterns will encapsulate information on the contextual factors, specific service touchpoints and associated resources, providing important input for how to prioritise improvements in care experiences.

***Emotion analysis and suggestion extraction.*** In order to identify and prioritise the key areas requiring improvements from patients’ and women’s perspectives, the affect and emotion expressed by patients and women who have given birth will be quantified. To estimate the emotion, we propose to use a linguistic analytics-based state anxiety detection algorithm in addition to traditional sentiment analysis. Our anxiety detection algorithm was trained on a dataset of 600 randomly selected tweets from 10,386 users ^32^, and scored by 604 zero-acquaintance human raters based on a six-item short-form of the Spielberger State-Trait Anxiety Inventory (STAI) ^33^. On average, each tweet was rated five times on a scale from 1 (“not at all”; low anxiety) to 4 (“very much”; high anxiety) by each rater. The training procedure involved the use of a 6-fold cross-validation resampling plan and was validated using a set of 3.33 million tweets. Using this algorithm has allowed us to account for the interaction between personality and anxiety in correspondence to the COVID-19 crisis ^34^, examine the influence of leaders over followers in organisations over time, and study public sentiment in response to organisational crisis communications on social media ^35, 36^.

**Figure 2.**
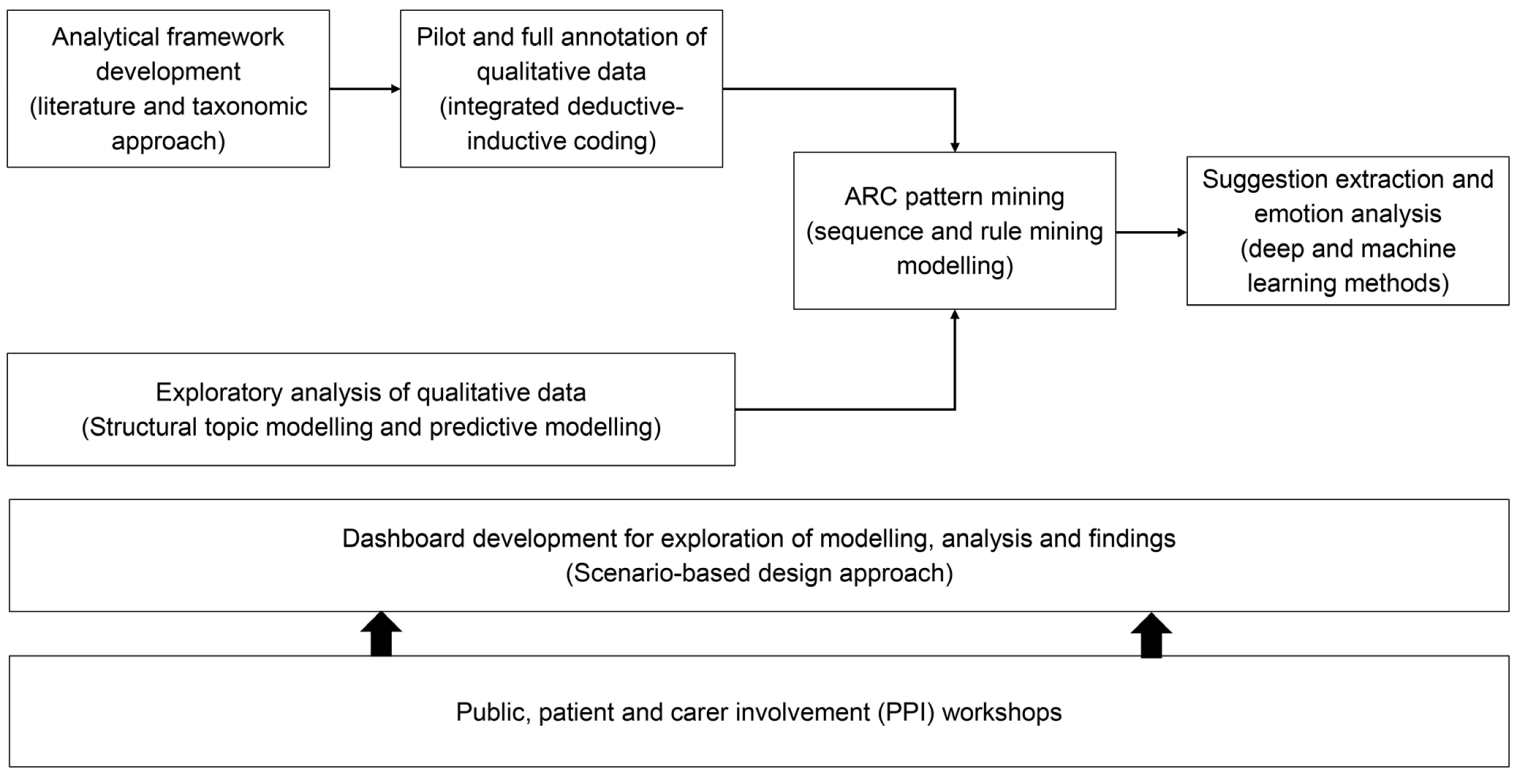
Research design and methodological approach.

To analyse the suggestions provided by patients as part of their qualitative feedback, we intend to use a deep learning model to automatically identify and classify comments where a suggestion is made. The automated approach is necessary given the volume of qualitative data, and will involve multi-label text classification, where each comment could be assigned one or more labels (topics of suggestion). We propose an end-to-end neural network architecture for multi-label classification of suggestion comments, and will use an existing training dataset for our machine learning task. Linking the extracted suggestions to the identified priority areas for improvement will facilitate the development of initiatives to improve inpatient and maternity care experiences. Figure 2 shows a summary of the study design and methodological approaches.

### Dashboard development

To provide knowledge users and other stakeholders with access to the results of our analyses, we will develop an analytics dashboard. The dashboard will enable the visualisation and exploration of findings with support to drill down to specific hospitals, hospital groups and practices within hospitals or specific themes such as safety, hygiene or ambulatory services. Development of the dashboard will involve iteratively developing scenarios for the use of the dashboard by different user groups, followed by elaboration of these scenarios into concrete information and decision needs for different groups. Finally, the information and decision needs will be translated into wireframe designs of the dashboard, and implemented using a robust analytics framework. 

### Patient and public involvement

Patients and carers will be involved throughout the conduct of the study. PPI representatives were invited to review and suggest amendments on the proposed research design, aims, and objectives. A patient, carer and public involvement (PPI) advisory group consisting of c. 5–10 patient representatives, family members, carers and the public will be involved in this study on an ongoing basis, including at key milestones, important strategic and methodological decisions. The PPI advisory group will attend project steering group meetings. Additional lay PPI representatives will be invited to participate through existing contacts and channels, as the National Care Experience Programme regularly consults with and involves lay patients, family members, carers and the public in the development of new surveys.

A number of formal engagement activities are planned in order to ensure continued and meaningful engagement. A collective intelligence session will allow PPI representatives to review the draft analytical framework and identify missing themes, ensuring participants can directly shape the analysis and insights to be produced from the secondary analysis.

Workshops will facilitate patient, carer and public input in the preliminary analysis and findings. PPI members and health service staff will engage in a co-design session of the analytics dashboard for exploration of findings. A final engagement will involve the presentation of the final results from the project, validation of the developed analytics dashboard through hands-on sessions for participants and obtaining input on the development of dissemination materials for the project. PPI members will help to inform and frame how findings from the surveys should be communicated in order to engage patients and carers. In organising these engagement workshops, we will carefully consider possible barriers to participation and address them to ensure engagement goals are realised.

### Ethics and dissemination

***Ethical considerations.*** This study involves secondary analysis of anonymised qualitative data from the National Inpatient Experience Survey and National Maternity Experience Survey. Ethical approval for the National Inpatient Experience Survey was granted by the Royal College of Physicians in Ireland (RCPI) Research Ethics Committee in March 2018, with approval updated on an annual basis thereafter. Ethical approval for the National Maternity Experience Survey was granted by the Royal College of Physicians in Ireland (RCPI) as an addendum to the ethical approval for the National Inpatient Experience Survey on 28 January 2019. While all qualitative data collected as part of these surveys are anonymised prior to the reporting of results by the National Care Experience Programme, there is a possibility that some potentially identifiable information (such as the names of hospital wards) may be present in the data. As part of the data management and governance tasks, the study team will re-screen the data to identify any mentions of names of people, specific ward etc. and remove them prior to further analysis. All PPI members will give informed, written consent prior to their involvement.

***Dissemination.*** Findings from this study will be disseminated through the publication of a national report, a webinar, press release, video animation, social media campaign and infographics for the general public, as well as publications in peer-reviewed journals and presentations at academic fora. Knowledge translation activities will include regular meetings of all project partners and development of quality improvement plans. This study will build upon the well-established partnership between the Irish Health Information and Quality Authority (HIQA), the Health Service Executive (HSE) and the Department of Health to ensure that senior HIQA, HSE and Department of Health stakeholders are accountable for responding to the findings. The involvement of knowledge users, stakeholder workshops and the development of eLearning tools will ensure purpose-built resources are in place to aid understanding of project findings, assist in the use of the newly developed dashboard and provide practical guidance on using the findings to improve areas where deficiencies in care experiences are highlighted.

## Study status

The study commenced on 1 April 2022, focusing initially on the smaller dataset from the National Maternity Experience Survey. Following an exploratory analysis of the maternity experience data, including structural topic models and predictive modelling, approximately 150 topics/themes were extracted from the free-text comments. These themes are currently being finalised, following which their relationship with demographic factors and overall experiences of care will be explored. A framework of the key activities, resources and context (ARC) factors associated with positive and negative maternity experiences is currently being developed. Once completed, the previously identified themes will be mapped onto the ARC framework, and the frequency of ARC patterns associated with the themes will be examined. The automatic annotation of maternity data has commenced to develop a deep learning model that automatically identifies ARC elements from women’s comments. Precision and recall scores will be used to gauge the usability of the model, with the aim of achieving 80% scores for both precision and recall. The extraction of linguistic rules from women’s comments is in progress, which will subsequently be used as a complementary technique for the automatic annotation of datasets. A similar approach will then be applied to data from the National Inpatient Experience Survey. Finally, plans and protocols for PPI activities are in place, and the recruitment process is in progress. The first PPI workshop is scheduled for October 2022, and will help to identify missing themes, refine the research questions, and to inform the theoretical lens and research approach.

## Conclusion

Considering the needs and preferences of service users in the planning, design and delivery of healthcare services can improve care experiences, which can in turn lead to improved outcomes, including better health and wellbeing. There is to date no unified approach to the analysis and integration of the detailed feedback received in response to the open-ended questions included on routinely conducted care experience surveys. This study aims to use the qualitative data collected as part of routine care experience surveys to their full potential, while also making this information easier to access and use by those involved in developing quality improvement initiatives. The study will include the development of a tool to facilitate more efficient and standardised analysis of care experience data on an ongoing basis, enhancing and accelerating the translation of patient experience data into quality improvement initiatives that address elements of patient experiences that have been highlighted by patients themselves as being important.

## Data Availability

No data are associated with this article.
