# Supplementary material for: Generating actionable insights from free-text care experience survey data using qualitative and computational text analysis: A study protocol
Source: HRB Open Res. 2022 Sep 12;5:60. [Version 1] doi: 10.12688/hrbopenres.13606.1 (PMC10663659; doi:10.12688/hrbopenres.13606.1)
Supplement: Peer review history

# Reviewer report: Reviewer response for version 1

**DOI:** 10.21956/hrbopenres.14869.r36820

**Published:** 2023-11-21

**Authors:** Emily O'Dowd

**Affiliations:** 1 Royal College of Surgeons in Ireland, Dublin, Ireland

This article outlines a protocol for a planned study on qualitative responses to the National Inpatient Experience Survey and National Maternity Survey.

 The authors have clearly presented their plans for the study, with a comprehensive rationale and detailed discussion of the methods to be used. While I am not an expert on the methods of analysis that they will employ, they seem to be well delineated and appropriate to address the aims. 

 Some further discussion on the boundaries between the comments that will be analysed, and formal complaints, would be interesting - though perhaps would be better placed in the study itself rather than the protocol. How do the authors plan to determine where the boundary is between a negative comment and a complaint? Do they need to? Some consideration of this would be interesting. 

 Overall, I'm very happy with the standard of work presented in this protocol and look forward to the study.

Is the study design appropriate for the research question?

Yes

Is the rationale for, and objectives of, the study clearly described?

Yes

Are sufficient details of the methods provided to allow replication by others?

Yes

Are the datasets clearly presented in a useable and accessible format?

Not applicable

Reviewer Expertise:

Complaints analysis, health services research, patient safety, human factors

I confirm that I have read this submission and believe that I have an appropriate level of expertise to confirm that it is of an acceptable scientific standard.

# Reviewer report: Reviewer response for version 1

**DOI:** 10.21956/hrbopenres.14869.r36201

**Published:** 2023-11-14

**Authors:** Chantal Arditi

**Affiliations:** 1 Center for Primary Care and Public Health (Unisanté), Department of Epidemiology and Health Systems, University of Lausanne, Lausanne, Vaud, Switzerland

The article presents the protocol of a study started in April 2022. The study aims to a) analyze large amounts of free text comments from two patient surveys, b) identify key areas for improvement from those comments, and c) develop a user-friendly dashboard for the results.

 This is important and useful research to make full use of all data provided in patient surveys, including free text comments. The article is well written and thorough. Many details are provided about the theorical framework as well as technical aspects. I applaud the authors for all the PPI activities planned in all steps of the study and the dissemination plan involving all stakeholders. However, a few aspects outlined below could be further clarified for the reader. 

1. The authors talk about 93,135 comments. Are they all in English? What about those written in other languages? It would be useful to know how the authors dealt with minority languages.
2. I’m guessing the comments from the 2 surveys will be analyzed separately; it would be useful to state this more clearly in the article.
3. Regarding the automated analysis using computational and machine learning techniques, I don’t have the expertise to comment on the technical aspects and validity of the methods proposed, but they seem valid and in line with what I’ve read. However, for those with the expertise, it could be useful to make the analytical tool available on an open-sourced platform like git-hub. In addition, sharing the tool would be a step towards the global aim of promoting a more unified approach to analyze free-text comments, which is missing as stated in the introduction. Do the authors plan to share the developed tool with other teams or researchers?
4. It would be useful for the reader to add some statistical references. In the paragraph entitled “structural topic models” consider adding a reference for your sentence “The Latent Dirichlet Allocation (LDA) technique is one of the most notable approaches for unsupervised topic modelling.” In the paragraph entitled “predictive modelling with random forest”, consider adding a reference for the Random Forest algorithm in the 2 ^nd^ sentence. In the paragraph starting with “ARC pattern mining” consider adding a reference to the Apriori algorithm.
5. The methods to analyze the free text data (objective 1) are presented in detail; the methods to identify key areas for improvement, policy development, healthcare regulation and monitoring, as well as possible quality improvement initiatives (objective 2) could be clarified. The authors mention “emotion analysis and suggestion extraction” using an anxiety detection algorithm. Has this been used before for this purpose? Is this the only method that will be used to identify key areas? If so, then it seems that only areas with strong anxiety will be identified and possibly targeted, which might put some important issues aside. What about areas inducing less anxiety but mentioned by many people? Would this be identified as a key area for improvement?

Is the study design appropriate for the research question?

Yes

Is the rationale for, and objectives of, the study clearly described?

Yes

Are sufficient details of the methods provided to allow replication by others?

Partly

Are the datasets clearly presented in a useable and accessible format?

Not applicable

Reviewer Expertise:

patient-reported experiences of care; public and patient involvement; free-text comments; quality of care; person-centered care; value-based care

I confirm that I have read this submission and believe that I have an appropriate level of expertise to confirm that it is of an acceptable scientific standard, however I have significant reservations, as outlined above.

# Reviewer report: Reviewer response for version 1

**DOI:** 10.21956/hrbopenres.14869.r33156

**Published:** 2022-11-14

**Authors:** Jenny King

**Affiliations:** 1 Picker Institute Europe, Oxford, UK

This project details work underway to make better use of free-text comments to experience surveys, including supporting organisations to use data for improvement. I have answered partly to 'Are sufficient details of the methods provided to allow replication by others?' as there are a couple of areas I would like to understand a bit more about.

1. Dealing with multiple themes within a comment. Will comments that cover multiple topics/sub-topics be broken into segments in outputs, with each segment receiving its own topic/sub-topic? Or are comments being dealt with as a whole?
2. Sentiment analysis is being carried out. I'd like to understand a bit more about how the researchers will take into account the different focus the three open ended questions have. In my experience sentiment analysis can sometimes be less effective where a question already has a positive or negative leaning. Is this being considered at all? As part of the sentiment analysis process, I'd expect there to be a risk that difficult sentence structures such as ones containing double negatives or sarcasm will be incorrectly assigned a sentiment value. Given volume of data this may be difficult to prevent, but would need to be a stated limitation in outputs produced.
3. Is the intention that the inpatient and maternity data will be processed as one, or will there be some allowance for differences in topics etc. that will be seen between the collections? Understanding this is important as differences between datasets will be starker for other collections this work might be expanded to (such as end of life care and nursing homes).

My last query is in relation to the mention of complaints data in the introduction. Whilst I understand why the link has been made here, by discussing complaints in the introduction it sets the reader up for thinking that the protocol will cover analysis of complaints data. Which isn't the case. Perhaps there could be a line added somewhere that notes that next steps/future work will look at complaints data too, if that is planned?

Is the study design appropriate for the research question?

Yes

Is the rationale for, and objectives of, the study clearly described?

Yes

Are sufficient details of the methods provided to allow replication by others?

Partly

Are the datasets clearly presented in a useable and accessible format?

Not applicable

Reviewer Expertise:

Person centred care, research methods, patient experience, staff experience, quantitative research, qualitative research.

I confirm that I have read this submission and believe that I have an appropriate level of expertise to confirm that it is of an acceptable scientific standard, however I have significant reservations, as outlined above.
